# To Be Continued

**Published:** 1884-02

**Authors:** 


### "To Be Continued."

Sylvanus Cobb used to write "a new story," and continue it from week to week, from month to month, and from year to year.

Every now and then we receive a dental journal with a "to be continued." Now, as a rule, we are opposed to "to be continued," and especially when it has but two or three pages of matter before it.

When a subject is printed and is to be read and studied, it is, as a rule, better to have the whole in hand.

We would not for a moment dictate how anybody shall conduct their journals, but we merely suggest that avoiding the "to be ——" in all cases when at all practicable will be acceptable to a great many readers. An article complete in one number will often be read when, if presented in sections, it will receive little or no attention. The better method is to present every article complete in one number, if it does not exceed twenty-five or thirty pages.

Paragraphs and items are popular, but everything can not be so presented.

